# Reply to: Unenhanced magnetic resonance imaging for the evaluation of sonographically indeterminate ovarian and adnexal masses

**DOI:** 10.1590/0100-3984.2025.0086

**Published:** 2025-10-15

**Authors:** Claudio Marcio Amaral de Oliveira Lima, Edson Marchiori, Antônio Carlos Coutinho Junior

**Affiliations:** 1 Centro de Diagnóstico por Imagem, Casa de Saúde N. Sra. de Fátima (Fatima Digittal), Nova Iguaçu, RJ, Brazil; 2 Departamento de Radiologia, Universidade Federal do Rio de Janeiro (UFRJ), Rio de Janeiro, RJ, Brazil; 3 Clínica de Diagnóstico por Imagem (CDPI), Rio de Janeiro, RJ, Brazil

Dear Editor,

We read with great interest the article by Moradi et al.^**(1)**^, which evaluates the diagnostic performance of unenhanced pelvic magnetic resonance imaging (MRI) in characterizing adnexal masses categorized as indeterminate on ultrasound. This timely study addresses an important clinical challenge, offering a safe and feasible protocol for patients in whom gadolinium-based contrast agents are contraindicated.

We congratulate the authors on their robust methodology, clear scoring system, and excellent diagnostic results-specificity of 97.7% and accuracy of 93.8%. These findings underscore the potential of unenhanced MRI as a valuable alternative in scenarios such as advanced chronic kidney disease, pregnancy, and patient refusal of contrast.

In the spirit of collaboration and building upon this important contribution, we would like to share some practical considerations that may further support the clinical applicability and integration of this strategy.

## False-positives in inflammatory and degenerative lesions

The misclassification of tubo-ovarian abscesses and degenerating fibroids as suspicious (score of 4 or 5) is understandable in the absence of contrast. However, ancillary features can aid differentiation. Fibroids often demonstrate a homogeneous low T2 signal even when central necrosis is present, whereas abscesses frequently show inflammatory fat stranding and perilesional edema, unusual in ovarian malignancy. Incorporating such elements, or creating a modified category for atypical benign patterns, may reduce overtreatment and patient anxiety. Clinical correlation continues to be essential^**(2)**^.

## Quantitative apparent diffusion coefficient analysis

The qualitative assessment of diffusion-weighted imaging used by the authors reflects expert practice, but interobserver variability remains a concern. Quantitative apparent diffusion coefficient (ADC) thresholds could improve reproducibility, especially in non-specialist settings. Values below ~1.0 × 10^-3^ mm^2^/s are more often associated with malignancy, whereas higher values generally favor benign disease. Systematic ADC quantification, validated across institutions and scanners, would strengthen the reliability of this score^**(3-5)**^.

## Future directions and validation

The excellent interobserver agreement reported (kappa = 0.9) attests to internal robustness. Nonetheless, external validation in general practice and multicenter prospective trials are crucial to confirm applicability in diverse populations. Integration with clinical and laboratory data, as well as standardized reporting across institutions, will be key to widespread adoption^**(3-5)**^.

## Conclusion

The study by Moradi et al.^**(1)**^ provides compelling evidence that unenhanced MRI can serve as an accurate alternative when contrast use is not feasible. With minor refinements-such as addressing benign mimics, incorporating quantitative diffusion analysis, and validating the strategy in broader practice-this diagnostic framework has the potential to meaningfully advance gynecologic oncology imaging and patient care. We thank the authors for their significant contribution and believe their work will stimulate further research and clinical innovation in this field.
